# Equity and vaccine uptake: a cross-sectional study of measles vaccination in Lasbela District, Pakistan

**DOI:** 10.1186/1472-698X-9-S1-S7

**Published:** 2009-10-14

**Authors:** Steven Mitchell, Neil Andersson, Noor Mohammad Ansari, Khalid Omer, José Legorreta Soberanis, Anne Cockcroft

**Affiliations:** 1 CIETcanada, 1 Stewart Street, Ottawa, Ontario, Canada; 2 Centro de Investigación de Enfermedades Tropicales (CIET), Universidad Autónoma de Guerrero, Acapulco, México; 3 CIET in Pakistan, House 226, Block 18, Gulshan-e-Iqbal, Karachi, Pakistan

## Abstract

**Background:**

Achieving equity means increased uptake of health services for those who need it most. But the poorest families continue to have the poorest service. In Pakistan, large numbers of children do not access vaccination against measles despite the national government's effort to achieve universal coverage.

**Methods:**

A cross-sectional study of a random sample of 23 rural and 9 urban communities in the Lasbela district of south Pakistan, explored knowledge, attitudes and discussion around measles vaccination. Several socioeconomic variables allowed examination of the role of inequities in vaccination uptake; 2479 mothers provided information about 4007 children aged 10 to 59 months. A Mantel-Haenszel stratification analysis, with and without adjustment for clustering, clarified determinants of measles vaccination in urban and rural areas.

**Results:**

A high proportion of mothers had appropriate knowledge of and positive attitudes to vaccination; many discussed vaccination, but only one half of children aged 10-59 months accessed vaccination. In urban areas, having an educated mother, discussing vaccinations, having correct knowledge about vaccinations, living in a community with a government vaccination facility within 5 km, and living in houses with better roofs were associated with vaccination uptake after adjusting for the effect of each of these variables and for clustering; maternal education was an equity factor even among those with good access. In rural areas, the combination of roof quality and access (vaccination post within 5 km) along with discussion about vaccines and knowledge about vaccines had an effect on uptake.

**Conclusion:**

Stagnating rates of vaccination coverage may be related to increasing inequities. A hopeful finding is that discussion about vaccines and knowledge about vaccines had a positive effect that was independent of the negative effect of inequity - in both urban and rural areas. At least as a short term strategy, there seems to be reason to expect an intervention increasing knowledge *and discussion *about vaccination in this district might increase uptake.

## Background

In health planning, a pro-equity approach requires the removal of obstacles to accessing services. However, inequities in many countries are increasing, leaving an ever widening gap between the rich and poor, and even dividing the poor into further gradients of vulnerability [1,2]. Expenditure on services is notoriously unbalanced, with the least vulnerable receiving the majority of investment [3]. This "poverty trap" means that the poorest and most vulnerable populations are less able to take up health care offers; this in turn worsens their socio-economic situation and health status [4,5].

Although there is debate about the definition of equity, there is a general consensus that health inequity constitutes inequalities in health that are unfair or unjust [6,7]. Rates of childhood vaccination are a good example. Vaccination is officially free in most countries, and in many developed countries its uptake is so close to universal as to have it considered "an indicator of how well children's rights are being respected" [8]. But vaccination coverage is lower in most developing countries, particularly in the poorest segments of these countries. Although vaccination is theoretically free, this does not account for costs of travel to the facilities and time away from work or the home. Poor access to facilities providing vaccination is a common reason for low uptake [9-11]. Other factors associated with reduced vaccination uptake include lack of maternal education [12], large family size [13], lack of household visits from health workers [14], and service provision issues such as a poor relationship between staff and clients and lack of trust that the vaccine is safe [15]. These disadvantages may be increased in vulnerable areas by, for example, water shortage, in comparison with which vaccination may not seem a pressing need [16]. Compounding reduced vaccination uptake, children from vulnerable households may have weaker immune systems and therefore be at increased risk of suffering severe consequences from measles [17]. In Bangladesh, for example, an unvaccinated child from a poor family in 2001 was more than twice as likely to die as an unvaccinated child from a family of higher economic status [18]. The costs of not vaccinating against measles have different implications for wealthier households for whom, with much less malnutrition and concomitant illness, childhood measles presents little more than an inconvenience [19].

Measles, a disease preventable by vaccination, primarily affects children in developing countries. According to the World Health Organisation (WHO) in 2001 there were over 30 million cases of measles and 777,000 deaths worldwide [20]. In Pakistan, estimates show that 20,000 children die from measles annually [21,22]. This is despite the Pakistan Expanded Programme on Immunization (EPI) which provides BCG, DPT, polio vaccine, and measles vaccine during the first year of a child's life. According to the Pakistan Ministry of Health, the programme is aiming for "90% routine immunization coverage of all EPI antigens with at least 80% coverage in every district by 2012" [23]. There is evidence suggesting that measles vaccination coverage has increased only slightly or even stagnated in some provinces in the last few years [24-26]. According to the Pakistan Social and Living Standards Measurement Survey (2006-07), for example, measles vaccination in Balochistan province fell from 70% in 2006 to 54% in 2007 [27].

To examine uptake of measles vaccination, we conducted a household survey in Lasbela, an impoverished district in the south-east corner of Balochistan province of Pakistan, in 2005. Using data from this survey, we have examined the factors related to measles vaccination uptake and in particular the role of inequities in determining this uptake.

## Methods

### Sample and data collection

At the time of the sample selection, Lasbela district consisted of five tehsils and had 22 union councils - 5 urban and 17 rural (the sampling and data collection took place before the addition of newly created tehsils, now totalling nine). We selected a stratified random cluster sample of communities to give representation of the situation in the different tehsils or talukas. First, union councils were randomly selected from each tehsil, reflecting urban/rural spread and with the number according to the population in each tehsil. We included a minimum of four union councils per tehsil to allow for tehsil level findings if needed for district level planning purposes. The official list of union councils provided by the district government was used as the sampling frame for the selection of union councils. From each union council we randomly selected one community (village or mohalla) from the list of communities in the union.

We drew a stratified random sample of 23 rural and 9 urban enumeration areas in Lasbela district to allow adequate representation of the heterogeneity across the district - particularly to allow tehsil level representation and urban/rural differentiation. In each selected community, the sample included a group of 100 contiguous households with children under five years, spreading out from a random starting point. There was no sampling within the site; all the eligible households were included.

Data collection instruments included a household questionnaire, which asked about household demographics and socio-economic status, and a questionnaire for the mothers or caregivers of children under 60 months old, which asked about the mothers' education, vaccine related knowledge, attitudes and practices, and about vaccination and illnesses of the children. The field teams also completed community profiles for each village of each community, by means of discussion with knowledgeable people and their own observations, including information about the location of facilities providing vaccination services and visits from mobile vaccination teams. It is possible for there to be different results within each community for this data as the community profiles were completed at the village level. For some indicators (i.e. visits by a vaccination team) there was missing data in the community profile for some villages, therefore reported denominators for these variables are smaller than variables from the household questionnaire.

Field teams comprising mainly women interviewers undertook the household survey in March and April 2005. After preliminary analysis of the household data, the teams returned to all of the sample communities in July 2005 and discussed the findings. Separate male and female focus group were conducted in each sample community [11].

### Analysis

Data entry used the public domain software package EpiInfo [28]; double data entry with validation reduced keystroke errors. Analysis relied on CIETmap open source software [29,30]. Although the sample drawn from each tehsil reflected its relative population size, this was not exact. To take into account under- and over-sampling of tehsils, we calculated population weights and applied these when making district level estimates. All the district level estimates reported in this article are weighted.

We examined associations between measles vaccination (among children aged 10-59 months), and related factors using the Mantel Haenszel procedure [31]. We first tested crude associations in a sequential analysis (stratifying by one factor at a time) and then used a multiple stratification - analogous to logistic regression analysis [32] - stepping down from an initial saturated model. Final results are presented as adjusted Odds Ratios (OR) and 95% confidence interval. Initial sequential stratification revealed that the associations between many of the variables and measles vaccination were different between urban and rural communities. We therefore built separate models for urban and rural settings.

In order to adjust for clustering, we applied Gilles Lamothe's robust variance estimator for cluster-correlated data to the Mantel-Haenszel stratification. Based on the odds ratio, the Lamothe estimator weights the effect rather than simply the in-cluster correlation. The adjustment works for medium and large data sets, where zero margins are not an issue.

Measurements of trend of vaccination uptake used the Mantel extension [33] calculated using the Statcalc module in Epi Info.

We used measles vaccination, as reported by the mother, as an indicator of vaccination coverage. In addition to uptake of measles vaccination as our primary outcome, we considered intermediate outcomes based on a behaviour change model called CASCADA, first developed in a study of HIV and AIDS prevention in 2001 [34] and subsequently used in developing an intervention to improve vaccination rates [24]. This model extends the knowledge, attitudes and practice model, adding more intermediate outcomes between knowledge and action. These include **c**onscious knowledge (able to correctly identify an illness preventable by vaccination), **a**ttitudes (think it is worthwhile to vaccinate), **s**ubjective norms (neighbours think it is worthwhile to vaccinate), intention to **c**hange (willing to take time away from daily activities to vaccinate), **a**gency (mother is involved in decisions about vaccination), discussion (discuss vaccinations within the household), and **a**ction (uptake of measles vaccination).

We defined several vulnerability variables to describe inequities between households and children that might be relevant to the uptake of measles vaccine.

#### Access

We divided children according to whether they lived within 5 km of a government facility offering vaccinations, and whether they lived in a community that was visited by a mobile vaccination team.

#### Type of roof

As a proxy for economic status we used roof quality, grouping roofs made of reinforced concrete, iron, asbestos or T-iron as good quality, and roofs that were thatched, mud or wood as poor quality.

#### Occupation of main breadwinner

Keeping in view the problems that are faced in asking directly about the household income, we used occupation of the main breadwinner as a proxy to the household economic status. We then grouped the households into those where the main breadwinner had an occupation with a potential of better yield in terms of income (such as skilled workers and office work) and those with a relatively poor occupation (such as unskilled worker or unemployed).

#### Household crowding

We calculated room occupancy by dividing the number of household members by the number of rooms in the household. We classed households with room occupancy of four or more as crowded.

#### Education of the mother

The education and literacy of women in Lasbela is low. We categorised mothers and caregivers according to whether they had *any *formal education or not.

#### Household visits from a lady health worker (LHW)

LHWs in Pakistan are an important source of preventive education and information for mothers. LHWs in Pakistan are considered as the prime source of preventive education and information to the households. They also counsel and motivate caregivers and household decision makers to immunize their children. We defined access to LHWs as mothers who had been visited by an LHW *and *who received information about vaccinations from the LHW.

#### Higher level variables

We generated higher level variables to test the combination of equity-related risk factors when these factors did not have a significant effect on their own. For example, in the rural multivariate model, we considered those who had the double disadvantage of poor access (further than 5 km from a government facility offering vaccination) and poor quality roofs.

## Results

The survey covered 3366 households in total, and reached 2479 mothers who provided information about 4007 children between 10 and 59 months of age.

### Vulnerability and equity factors

Less than one half (45%, 1846/3739) of children aged 10-59 months lived within 5 km of a government health facility offering vaccination services, and just over one-third (1361/3580) lived in areas visited by a vaccination team. Some 37% (1453/3989) of children aged 10-59 months lived in houses with good quality roofs; 51% (2034/3957) lived in houses where the main breadwinner had an occupation with better income; 41% (1610/3987) lived in less crowded households and 31% (1274/4007) lived in urban settings. Only 7% (291/4004) of children aged 10-59 months had mothers with any formal education. And only 8% (323/3981) had mothers that had been visited by a LHW and told about vaccinations.

Table 1 shows the different equity variables in urban and rural areas. More children in urban areas had mothers with some formal education (p < 0.001). The proportion of children with access to government vaccination facilities within 5 km was much higher in urban areas than in rural areas (p < 0.001). Vaccination team visits were higher in rural areas (p < 0.001), although still less than one half of the rural children lived in communities visited by a vaccination team. The proportion of rural children whose mothers received visits and information about vaccinations from an LHW was low; only 2% of mothers received this information from an LHW.

**Table 1:** Equity indicators among children aged 10-59 months.

	Percent (number)		
		
	Rural areas	Urban areas	p value
Mother with any formal education	3 (92/2730)	16 (199/1274)	<0.001
Live within 5 km of government vaccination facility	27 (815/2518)	84 (1031/1221)	<0.001
Live in community visited by a vaccination team	46 (1082/2328)	22 (279/1252)	<0.001
Mother visited by a LHW who talked about vaccinations	2 (80/2723)	19 (243/1258)	<0.001
Live in household with better roofs	25 (666/2728)	65 (787/1261)	<0.001
Live in household with better occupation of main breadwinner	50 (1383/2711)	53 (651/1246)	0.471
Live in less crowded household	37 (1013/2726)	49 (597/1261)	p < 0.001

### Knowledge, attitudes and discussion about vaccination

Knowledge about vaccinations was high. Some 86% (2164/2474) of mothers had heard about vaccinations and 76% (1884/2438) could correctly mention an illness that could be prevented by vaccination. Only 3% of mothers (74/2437) had heard something about bad effects of vaccinations. Nearly all (91%, 2255/2450) mothers felt it was worthwhile to vaccinate children. Among those with knowledge about vaccinations, even more (98%, 1841/1881) felt it was worthwhile to vaccinate.

Most (82%, 2046/2451) mothers felt their neighbours would agree that it was worthwhile to vaccinate children. Among those who did not say this, many (296) said they did not know how their neighbours felt. Nearly all (94%, 1989/2092) mothers said they would be willing to take some time out of their day to take a child from their household to be vaccinated. Most (87%, 2151/2469) mothers reported they were involved in decisions about vaccination, and most (85%, 2090/2429) mothers had discussed vaccination within the family. Knowledge, positive attitudes and rates of discussion were higher in urban areas than in rural areas (Table 2).

**Table 2:** Knowledge, attitudes and discussion of vaccination among mothers of children aged 10-59 months.

	Percent (number)		
		
	Rural areas	Urban areas	p value
Could correctly identify an illness preventable by vaccination	70 (1223/1692)	89 (661/746)	<0.001
Felt vaccinations were worthwhile	88 (1520/1694)	97 (735/756)	<0.001
Believed neighbours thought vaccinations were worthwhile	77 (1347/1695)	92 (699/756)	<0.001
Willing to take time to have child vaccinated	92 (1374/1468)	98 (615/624)	<0.001
Involved in decision about vaccinations	89 (1527/1715)	82 (624/754)	<0.001
Discussed vaccinations within the family	82 (1394/1676)	92 (696/753)	<0.001

### Inequity and measles vaccination uptake

Among children aged 12-23 months, slightly more than half (51%, 477/904) had received measles vaccine. Similarly, 51% (2103/3964) of the children aged 10-59 months had received the measles vaccine.

Tables 3 and 4 show the percentage of children aged 10-59 months vaccinated in urban and rural areas by different equity indicators. Notable is the fact that even among those in better socio-economic situations, in most cases only two-thirds of children are immunised. For example, in urban areas located within 5 km of a government vaccination facility, 68% (692/1023) of children aged 10-59 months are vaccinated. Similarly, among children in urban areas where the main breadwinner has a good job, 67% (433/643) are vaccinated. One exception to this is children from urban areas whose mothers' are educated (87% - 173/199).

**Table 3:** Measles vaccination among children aged 10-59 months (urban areas).

	Percent (number vaccinated)	p value
Males	64% (410/636)	p = 0.724
Females	63% (374/589)	
Live within 5 km of vaccination facility	68% (692/1023)	p < 0.001
Live further than 5 km of vaccination facility	44% (91/190)	
Visited by vaccination team	66% (182/277)	p = 0.575
Never visited by vaccination team	63% (617/966)	
Better roofs	66% (522/779)	p = 0.007
Poor roofs	59% (281/473)	
Better job	67% (433/643)	p = 0.007
Poor job	59% (356/594)	
Educated mother	87% (173/199)	p < 0.001
Non-educated mother	59% (636/1065)	

**Table 4:** Measles vaccination among children aged 10-59 months (rural areas).

	Percent (number vaccinated)	p value
Males	47% (672/1350)	p = 0.053
Females	43% (617/1340)	
Live within 5 km of vaccination facility	66% (551/811)	p < 0.001
Live further than 5 km of vaccination facility	38% (668/1674)	
Visited by vaccination team	51% (552/1064)	p < 0.001
Never visited by vaccination team	38% (521/1232)	
Better roofs	57% (383/660)	p < 0.001
Poor roofs	41% (908/2035)	
Better job	49% (706/1374)	p < 0.001
Poor job	41% (575/1304)	
Educated mother	62% (59/92)	p = 0.002
Non-educated mother	44% (1234/2605)	

Table 5 shows the variables included in the multivariate analysis, to further investigate the role of equity and other behavioural indicators in vaccination uptake of children aged 10-59 months. Table 6 shows the final model of the multivariate analysis for children living in *urban *areas. Having a mother with some education, who had discussed vaccinations, who knew of at least one vaccine-preventable illness, living in a community within 5 km of a government vaccination facility, being visited by a LHW who talked about vaccinations, and living in a house with a good roof were associated with vaccination uptake.

**Table 5:** Variables included in multivariate analysis of measles vaccination uptake among children aged 10-59 months.

Outcome:	Measles vaccination
Covariants:	Roof type
	Occupation
	Crowding
	Education of mother
	Rural/urban setting
	Sex of the child
	Mother could correctly identify an illness preventable by
	vaccination
	Mother discussed vaccination within the family
	Mother involved in decisions about child's vaccinations
	Mother visited by LHW and told about vaccinations
	Government vaccination facility within 5 km of area
	Community visited by a mobile vaccination team

**Table 6:** Multivariate model of factors associated with measles vaccination in urban areas.

Variable	Unadjusted OR	Adjusted OR	95%CI for adjusted OR	Cluster adjusted 95% CI for OR
Education of mother	4.46	3.59	2.33-5.55	**2.14-6.03**
Discussed vaccinations	3.80	3.13	2.04-4.82	**1.47-6.69**
Knowledge about vaccinations	3.71	2.57	1.74-3.82	**1.67-3.97**
Govt vaccination facility <5 km	2.45	1.95	1.37-2.78	**1.63-2.34**
Good roof	1.41	1.47	1.12-1.93	**1.08-2.01**
Access to LHW	1.79	1.76	1.22-2.53	0.90-3.43

Table 7 shows the final model of factors for children living in *rural *areas. The model included many of the same variables as in urban areas, with access to vaccination and roof of dwelling having a combined effect, where the individual effects were statistically insignificant.

**Table 7:** Multivariate model of variables associated with measles vaccination in rural areas.

Variable	Unadjusted OR	Adjusted OR	95%CI for adjusted OR	Cluster adjusted 95% CI for OR
Discussed vaccinations	6.42	4.17	3.10-5.61	1.19-14.64
Knowledge about vaccinations	3.50	2.22	1.78-2.78	1.36-3.63
Government vaccination <5 km and good roof	3.19	3.7	2.41-5.19	1.85-5.64

**Figure 1:**
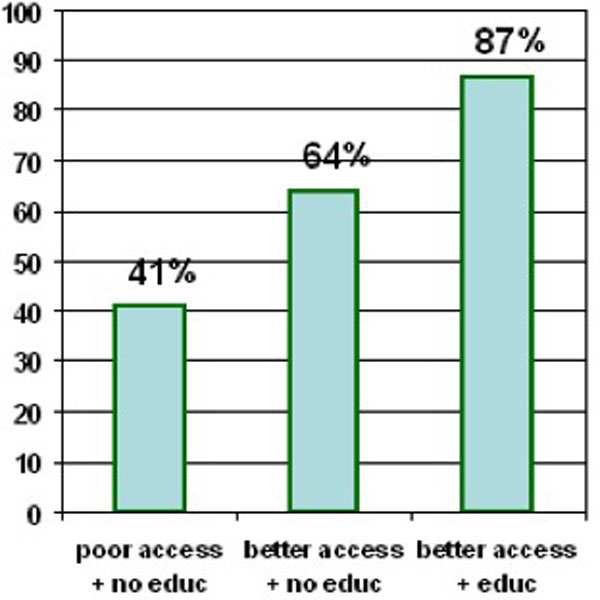
**Proportion of children aged 10-59 months vaccinated among equity sub-groups in urban areas**.

Figure 1 illustrates the compounding effects of inequities in urban areas, showing the two most prominent equity factors that resulted from the urban multivariate analysis model - education and access. There is a significant trend for increased vaccination as inequities are removed (Chi square for linear trend 72.510, p < 0.000). Among children living in households more than 5 km from a government facility providing vaccinations (poor vaccination access) and whose mother had no education, just 41% (75/173) had received measles vaccine. Among children with poor vaccination access and with mothers with some education, 64% (541/850) had received measles vaccine. Among children with the advantages of both better access to a vaccination facility and a mother with some education, 87% (151/173) had received measles vaccine. There were too few children with poor vaccination access whose mothers had any education to include this category in the figure.

**Figure 2:**
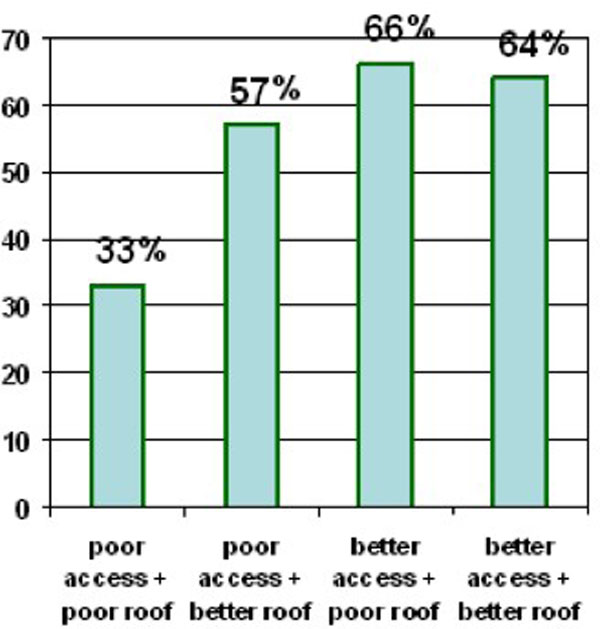
**Proportion of children aged 10-59 months vaccinated among equity sub-groups in rural areas**.

Figure 2 illustrates the compounding effects of inequities in rural areas, showing two prominent equity factors that resulted from the rural multivariate analysis model - access and roof type. Again, there is a significant trend for increased vaccination as inequities are removed (Chi square for linear trend 204.001, p < 0.000). Among children with poor vaccination access, those living in houses with a better roof were more likely to be vaccinated than those in houses with a poor roof (57% - 207/354 vs. 33% - 461/1320). Children with better vaccination access had higher rates of vaccination, and in this group there was little difference between those with a poor roof (66% (422/611) vaccinated) and those with a better roof (64% (129/200) vaccinated).

### Views of community focus groups

When they heard about the low rates of measles vaccination in their area and discussed the reasons for this, most of the focus groups, both male (24/32) and female (23/32), stressed the problems of access to services. Some (17/32 males focus groups and 13/32 female focus groups) noted that people were ignorant or had misconceptions about vaccination, for example: "*Some people say that in earlier times children were never vaccinated, but they still managed to survive*" (Female focus group).

Yet the groups noted that immediate financial and time costs associated with having a child vaccinated weigh very heavily in comparison with the potential costs associated with measles in the future. Poverty was mentioned by 23/32 male focus groups and 25/32 female focus groups as a major reason why children weren't vaccinated. The focus group participants were clear that poverty was a major limitation to vaccination in the context of the limited access to vaccination services in Lasbela. Overwhelmingly, their suggestions for how to increase vaccinations were concerned with increasing access to services. For example: "*Vaccinations must be available at all basic health units so that more children may be vaccinated.*" (Male focus group) and "*Teams must come here once every month and immunise our children." *(Female focus group)

## Discussion

Measles coverage in Lasbela is unacceptably low, with only one half of the children aged 12-23 months old vaccinated. This is despite most mothers knowing of a vaccine preventable illness, considering vaccination worthwhile, and discussing vaccinations within the family. We found a strong association between discussion of vaccination and vaccination status in both urban and rural areas. Yet discussion is clearly not enough; vaccination rates in Lasbela are low even among the high proportion of mothers who had discussed vaccination in the family.

Among the limitations of this study is the reliance on vaccination as reported by the mother, as an indicator of vaccination uptake. Some developed country authors have suggested that maternal recall is not a good enough indicator of vaccination status compared with health facility records [20,21]. However, a study from Italy found that parental recall alone was similar to other measures of vaccination status and concluded that "verbal recall should be accepted as reasonably reliable in the absence of cards" [22], while in Australia parental recall of measles vaccination coincided as well as vaccination cards with the presence of antibodies [23]. In Turkey, taking polio antibodies as the 'gold standard', a study found that parental recall was more sensitive but less specific than official records [24]. A study in India found that maternal recall underestimated children's vaccination status but using vaccination cards was not helpful because less than half the mothers had cards and the cards were often incomplete or grossly inaccurate [25]. Our own experience in Pakistan is that vaccination cards are frequently missing or highly inaccurate. Valadez et al in Costa Rica concluded that maternal recall could be used for estimating vaccination status, especially for younger children and for single dose vaccines [26]. Langsten and Hill in rural Egypt found mothers' reports were later confirmed by card data for at least 83% of children aged 12-23 months [27]. Gareaballah and Loevinsohn found that mothers' reports in the Sudan were accurate and concluded that for both DPT and measles vaccination, reliance on mothers' reports alone gave accurate estimates of vaccination coverage [28]. Goldman and Pebley in Guatemala highlighted the serious problems with service-based data (including vaccination cards) and recommended using mothers' reports to improve estimates of vaccination coverage [29]. Importantly, authors have reported that even if maternal recall may under- or over-estimate vaccination status, this was not related to factors such as maternal education level or poverty status [25,30]. We therefore believe that our reliance on maternal recall of vaccination status is reasonable and is not likely to have introduced bias into the analysis of factors related to vaccination in the Lasbela district.

Our findings illustrate the role of equity in determining vaccination uptake in Lasbela. In both urban and rural areas, access to a government facility providing vaccinations, a key equity factor, was a determining factor for uptake. This is consistent with other reports that identify poor access to vaccination services as an obstacle to uptake [9,10]. This was also confirmed during community feedback focus groups where many participants claimed their main obstacle to vaccinating their children was access to the facilities. In Lasbela, the proportion of children in rural areas with access to government facilities providing vaccination services is much lower than in urban areas and it is not universal even in urban areas.

In Lasbela we did *not *find visits by mobile vaccination teams associated with increased vaccination in either urban or rural areas. This is in contrast with the findings in other districts of Pakistan, where visits by vaccination teams were associated with increased measles vaccination, particularly in rural areas [35]. When asked how vaccinations might be increased, nearly all of the focus groups (29/32 male and 25/32 female) suggested *door-to-door *visits by the vaccination teams. It is likely that in Lasbela, where the terrain is mountainous and difficult to traverse, even when vaccination teams supposedly visit the community they are not reaching the most remote households in the communities. There could also be issues around service delivery (such as service provider attitudes, unofficial payments) that restrict the effectiveness of these initiatives.

In urban areas of Lasbela, where access to vaccination services is better than in rural areas, maternal education also played an important role in determining vaccine uptake, consistent with the findings of other authors [12,35]. However, we did not find that mother's education was related to vaccine uptake in rural areas, although the small number of mothers in these areas with formal education might explain this. We did find that indicators of better socio-economic status, such as good roof type and having a better job were important determinants of vaccine uptake. The focus groups confirmed the importance of costs (such as travel and time away from work) as obstacles to vaccination for poor families. These costs get higher as the distance to the facility increases, compounding inequity for those who are poorest.

Figures 1 and 2 illustrate the importance of different aspects of equity in determining vaccine uptake in urban and rural areas. In urban areas (Figure 1) better access to vaccination and maternal education seem to increase vaccination uptake by about the same amount, and maternal education compounds with access when it is present. This illustrates how, in urban areas with good access, inequities between households still exist, in this case in terms of maternal education. This is consistent with authors who believe that inequities are not limited only to rural and marginal areas, and can exist within all socioeconomic groups [2,36]. It also demonstrates that in urban areas at least, access alone is not the only equity factor involved in uptake of vaccination.

In rural areas, however, (Figure 2) there is a clear advantage for those living in areas with better access. The advantage for better off households (those with a better roof) seems to be confined to areas with poor access to services. It is probable that in areas with poor access the costs of taking a child for vaccination are much higher and therefore the disadvantage of poor households is more apparent. This compounding of inequity results in the very low vaccination rate of only 33% among poor rural households with poor access to services. This supports existing research that shows, while inequity is not *limited *to the most marginalised communities, the equity gap is increasing between the rich and the poor and that the poorest continue to receive the poorest service [1,37,38]. Overall rates of vaccination are lower in rural areas than in urban areas, and even lower still in the most marginalised rural areas.

Measles vaccination coverage is stagnating, or even decreasing, in some parts of Pakistan and this could be related to increasing inequities. Even when overall vaccination coverage in a country is increasing, this may mask considerable and even increasing inequities in coverage, particularly among the most vulnerable households [39]. Measures of vaccination coverage should include an assessment of inequities. The importance of different measures of inequity will vary from place to place, and even within different regions of one district. Understanding the particular dynamics of inequity and how it interacts with other factors related to vaccine uptake is a step towards increasing equity in vaccine coverage.

## Conclusion

Inequities of access, maternal education and household socio-economic status are important determinants of childhood measles vaccination uptake in a poor district of Pakistan with limited provision of vaccination services. These inequities compound one another, so children from families with multiple disadvantages are very unlikely to be vaccinated, marginalising them even further with higher risk of poor health.

A hopeful finding is that discussion about vaccines and knowledge about vaccines had a positive effect that was independent of the negative effect of inequity - in both urban and rural areas. At least as a short-term strategy, there seems to be reason to expect an intervention increasing knowledge *and discussion *about vaccination in this district might increase uptake.

## List of abbreviations used

WHO: World Health Organization; CIET: Community Information for Empowerment and Transparency; EPI: Expanded Programme on Immunisation; LHW: Lady Health Worker.

## Competing interests

The authors declare that they have no competing interests.

## Authors' contributions

SM contributed to the design, conducted the analysis and drafted the manuscript. NA designed the study, developed the methodology, and contributed to the analysis and the drafting of the manuscript. NMA contributed to the instrument design, supervised the fieldwork and contributed to data analysis. KO coordinated the project in Pakistan and contributed to the drafting of the manuscript. JLS contributed to the design, analysis and drafting of the manuscript. AC contributed to data analysis, was responsible for overseeing the project in Pakistan, and contributed to the drafting of the manuscript.
